# Mechanisms underlying the endogenous dopaminergic inhibition of spinal locomotor circuit function in *Xenopus* tadpoles

**DOI:** 10.1038/srep35749

**Published:** 2016-10-20

**Authors:** Laurence D. Picton, Keith T. Sillar

**Affiliations:** 1 School of Psychology and Neuroscience, University of St Andrews, St Andrews KY16 9JP, United Kingdom

## Abstract

Dopamine plays important roles in the development and modulation of motor control circuits. Here we show that dopamine exerts potent effects on the central pattern generator circuit controlling locomotory swimming in post-embryonic *Xenopus* tadpoles. Dopamine (0.5–100 μM) reduced fictive swim bout occurrence and caused both spontaneous and evoked episodes to become shorter, slower and weaker. The D2-like receptor agonist quinpirole mimicked this repertoire of inhibitory effects on swimming, whilst the D4 receptor antagonist, L745,870, had the opposite effects. The dopamine reuptake inhibitor bupropion potently inhibited fictive swimming, demonstrating that dopamine constitutes an endogenous modulatory system. Both dopamine and quinpirole also inhibited swimming in spinalised preparations, suggesting spinally located dopamine receptors. Dopamine and quinpirole hyperpolarised identified rhythmically active spinal neurons, increased rheobase and reduced spike probability both during swimming and in response to current injection. The hyperpolarisation was TTX-resistant and was accompanied by decreased input resistance, suggesting that dopamine opens a K^+^ channel. The K^+^ channel blocker barium chloride (but not TEA, glybenclamide or tertiapin-Q) significantly occluded the hyperpolarisation. Overall, we show that endogenously released dopamine acts upon spinally located D2-like receptors, leading to a rapid inhibitory modulation of swimming via the opening of a K^+^ channel.

Neural circuits of the spinal cord that generate rhythmic locomotor activity, so called central pattern generators (CPGs), are subject to profound modulatory influences which alter neuronal properties and synaptic strengths to confer flexibility on locomotor output and behaviour^1^. Sources of neuromodulators are present both intrinsically within the spinal cord and extrinsically in higher centres of the brainstem (for a recent review see ref. 2). The neuromodulators involved comprise a heterogeneous list of signalling molecules including classical neurotransmitters, peptides, biogenic amines and the gaseous free radical nitric oxide. The biogenic amines serotonin, noradrenaline and dopamine, emanating primarily from brainstem nuclei, form diffuse modulatory systems that project to spinal sensory and motor circuits and are known to modulate CPG output. The majority of neuromodulators function via changes in intracellular 2^nd^ messenger concentrations following activation of G protein coupled receptors (GPCRs) and many neuromodulators exert their influences by activation of more than one pharmacologically distinct subset of GPCRs with distinct physiological actions. Dopamine, for example, acts upon two principal classes of receptor which generally exert inhibitory (D2-like: D2,D3,D4) or excitatory (D1-like: D1,D5) influences on neural circuits^3^.

Dopaminergic fibres forming descending tracts from the brain offer a rich source of neuromodulatory input to the axial locomotor circuits in the spinal cords of aquatic vertebrates (lamprey^4^ and zebrafish^5^); amphibian tadpoles^6^, and the appendicular spinal circuits of mammals^7^. The diencephalic dopaminergic tract (DDT) forms a phylogenetically conserved descending projection which is present in all vertebrates studied to date and provides the earliest developing aminergic projection to the spinal cord^8^. In zebrafish, endogenous dopamine plays important roles in establishing the complement of CPG neurons early in development^9^. Dopamine also subsequently controls the developmental expression of motor pattern generation by acting on a range of dopamine receptors^5^,^10^,^11^. In free-swimming larval *Xenopus laevis* tadpoles, dopamine has opposing, concentration-dependent effects on swimming: low dopamine concentrations activate high affinity D2-like receptors to inhibit spontaneous locomotor activity; while high dopamine concentrations facilitate locomotion by activating lower affinity D1-like receptors^6^. However, this previous research on older, pre-metamorphic free swimming stages of tadpole development was obtained using extracellular ventral root recordings and did not address the cellular mechanisms responsible for dopamine’s actions in the spinal cord or the developmental onset of the dopaminergic system.

In the present study we have examined the role of dopamine at earlier stages of *Xenopus* development, around the time of hatching, when the animal possesses a simpler and much better defined spinal locomotor circuit^12^. Neurons immunoreactive for tyrosine hydroxylase (TH) are known to be present in the mesencephalic posterior tuberculum (PT), the spinal cord and other areas of the CNS at these early stages of development^13^, but the role of the dopaminergic system at these stages remains unknown. Our results reveal a potent inhibitory role for dopamine at these early developmental stages, suggesting a preponderance of D2-like receptors and a paucity of D1-like receptors early in development. The mechanism appears to involve the endogenous activation of spinally located D2-like receptors, which leads to the hyperpolarisation of rhythm-generating neurons of the swim CPG via the opening of a K^+^ channel.

## Results

### Dopamine inhibits the parameters of fictive swimming

**Figure 1:**
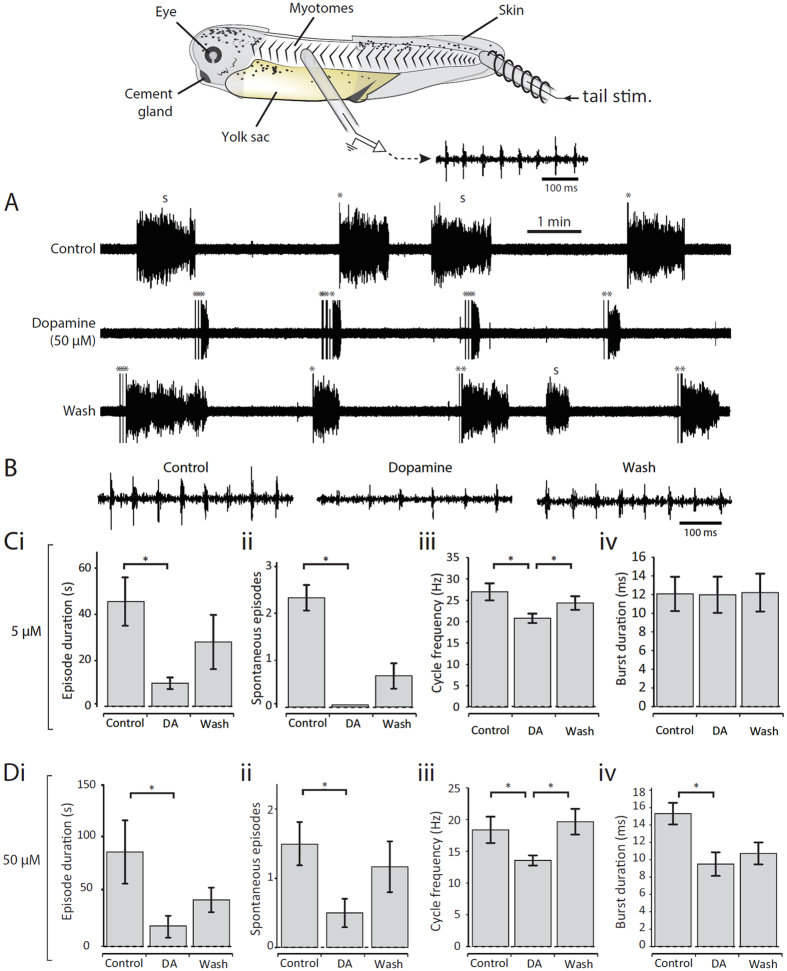
Dopamine inhibits fictive swimming across a range of concentrations. Top: The experimental preparation. (**A**) Raw traces showing evoked and spontaneous episodes of fictive swimming in control, in the presence of 50 μM dopamine and after dopamine wash-out. “s” denotes spontaneous swim episodes and “*” denotes tail stimulus. (**B**) Raw traces showing 500 ms of activity at the start of an evoked episode in control, in dopamine (50 μM) and after dopamine washout. Note that the swimming is slower and weaker in dopamine compared to control and wash. (**C**) A low concentration of dopamine (5 μM) significantly reduced episode duration (i: p < 0.05, n = 6); the number of spontaneous episodes of swimming (ii: p < 0.05, n = 3); and swim frequency (iii: p < 0.05, n = 6). (**D**) A high concentration of dopamine (50 μM) was also inhibitory and significantly reduced episode duration (i: p < 0.05, n = 8); the number of spontaneous episodes of swimming (ii: p < 0.05, n = 6); swim frequency (iii: p < 0.05, n = 8); and burst durations (ii; p < 0.05, n = 5).

In an initial set of experiments, dopamine was bath-applied at a range of concentrations to immobilized *Xenopus* tadpoles to assess its modulatory influence on spontaneous and electrically evoked episodes of fictive swimming. In keeping with a previous report on older free swimming *Xenopus* tadpoles^6^, low concentrations of dopamine (0.5–5 μM) reduced the probability of occurrence of spontaneous swimming episodes (Fig. 1Cii). In addition, and in contrast to older stages, several parameters of both spontaneous and evoked fictive swimming were also reduced in the presence of a low dose of dopamine including swim episode duration (Fig. 1Ci) and cycle period (Fig. 1B,Ciii). These effects of dopamine were partially reversible after approximately 30 mins return to control saline, although this only reached statistical significance for swim cycle frequency. Next, higher dopamine concentrations (50–100 μM) were applied to address whether, as in older tadpole stages, the effects of dopamine switch to become excitatory at high doses. However, only the inhibitory effects of dopamine were observed (Fig. 1A,Di–v), and even exaggerated at the higher doses, suggesting that a D2-like effect alone is present at these early stages of development. Compared to other modulators in this preparation, such as serotonin and noradrenaline^14^, the onset of the effects of dopamine on swimming activity occurred extremely rapidly (<1 minute after entering the bath). In addition to effects on the parameters of swimming, dopamine also increased the sensory threshold for evoking swimming, as evidenced by the increase in the number and intensity of stimulation pulses needed to trigger swimming in Fig. 1A, middle trace. However, this aspect of dopamine modulation was not studied in more detail here.

### Dopamine inhibition is mediated via D2-like receptors in the spinal cord

**Figure 2:**
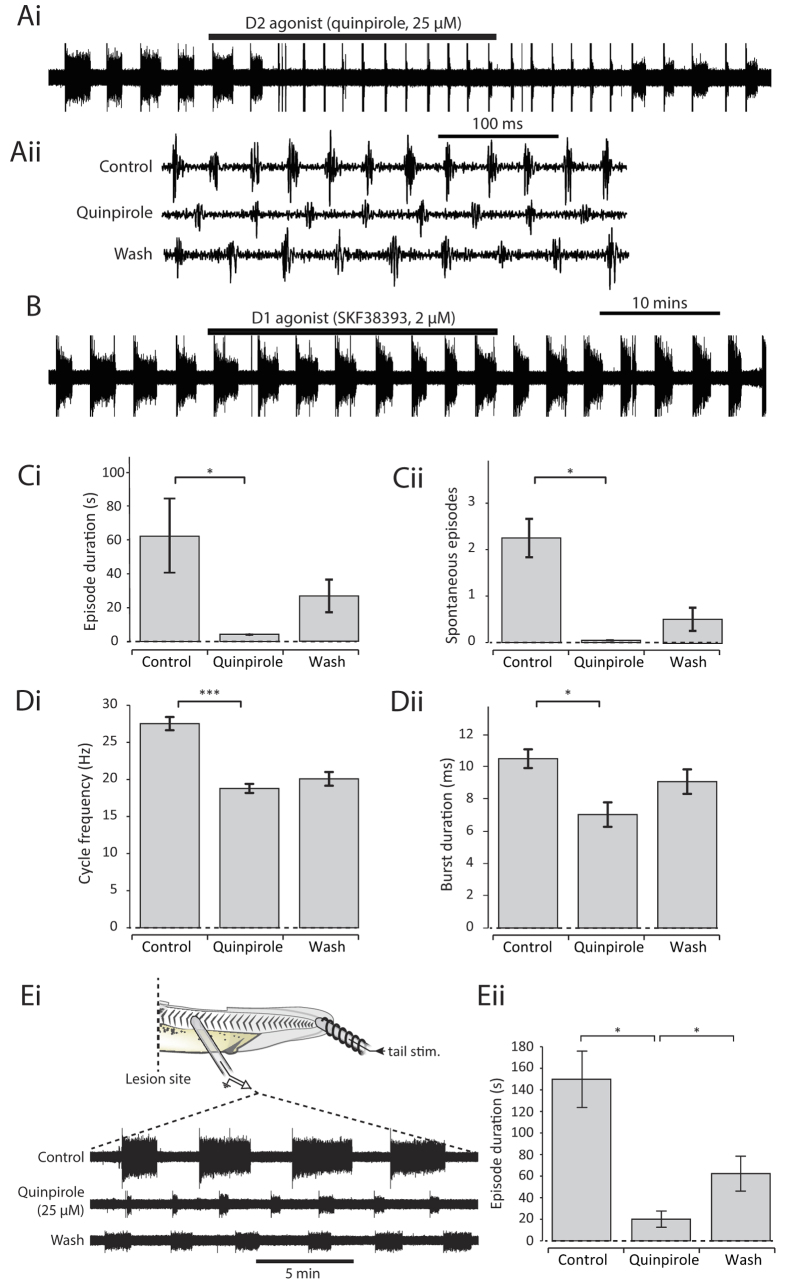
The inhibitory effects of dopamine on the swim network are mediated via spinal D2-like receptors. (**Ai**) Raw trace showing the effect of the D2-like agonist quinpirole (25 μM) on fictive swimming. (**Aii**) Raw trace showing a 500 ms excerpt of activity in control, in the presence of quinpirole, and after washout. (**B**) Raw trace showing the lack of effects of the D1-like agonist SKF38393 (2 μM). (**C**) Quinpirole (25 μM) significantly reduced episode duration (i: p < 0.05, n = 9) and the number of spontaneous episodes of swimming (ii: p < 0.05, n = 4). (**D**) Quinpirole (25 μM) also significantly reduced swim frequency (i: p < 0.001, n = 9) and burst durations (ii; p < 0.05, n = 6). (**E**) Quinpirole (25 μM) still had an inhibitory effect in tadpoles that had been spinalised at the level of the 2nd post-otic intermyotomal cleft, suggesting that inhibitory D2-like receptors are present in the spinal cord.

Dopamine acts upon two principal classes of receptor which generally exert inhibitory (D2-like: D2,D3,D4) or excitatory (D1-like: D1,D5) influences on neural circuits. To confirm that the inhibitory effects of dopamine on the *Xenopus* swim circuit are indeed mediated via actions at D2-like receptors, the broad-spectrum D2-like agonist quinpirole (25 μM) was bath applied. Quinpirole faithfully mimicked all of the effects of dopamine by rapidly reducing the occurrence of spontaneous episodes of fictive swimming and inhibiting the same parameters of motor output including cycle period, burst duration and burst amplitude (Fig. 2). The fact that quinpirole and dopamine effects were qualitatively the same suggests that the effects of dopamine are exclusively mediated at D2-like receptors at this early stage of development. This conclusion is supported by the fact that the D1 agonist SKF38393 (2 μM) exerted no significant effects on any of the parameters of the fictive swimming rhythm at these early stages of development (Fig. 2B).

In order to investigate whether the D2-like receptors mediating inhibitory effects on the tadpole swim CPG are located in the spinal cord and/or in higher brain centres, the immobilized preparation was spinalized at the level of the 2^nd^ post-otic intermyotomal cleft (Fig. 2Ei). This procedure leaves sufficient rhythm generating spinal circuitry in the spinal cord to produce sustained swim episodes in response to skin stimulation^15^, but removes the influence of descending brainstem pathways, abolishes spontaneous episodes and regularizes swim episode parameters. Quinpirole (25 μM) continued to exert as profound an inhibitory effect on fictive swimming as intact preparations (Fig. 2Ei,ii), indicating that the D2-like receptors involved in dopamine modulation are located on CPG neurons of the spinal cord.

### Endogenously released dopamine inhibits swimming

**Figure 3:**
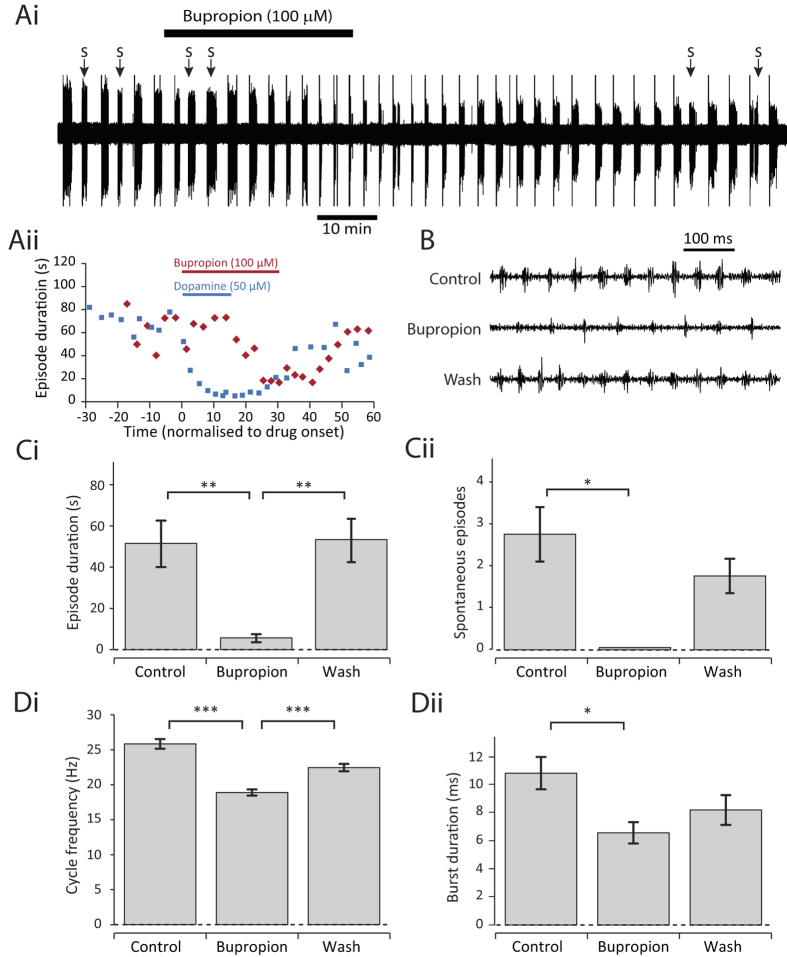
Endogenously released dopamine inhibits swimming. (**Ai**) Raw trace of fictive swimming showing an entire 2 hour experiment. The application of 100 μM bupropion clearly inhibited swimming after approximately 20 minutes. (**Aii**) A comparison of the effect onset in a bupropion experiment and a dopamine experiment. (**B**) Raw traces showing 500 ms of activity at the start of an evoked episode in control, 100 μM bupropion, and following drug washout. (**C**) Bupropion (100 μM) significantly reduced episode duration (i: p < 0.01, n = 13) and the number of spontaneous episodes of swimming (ii: p < 0.05, n = 4). (**D**) Bupropion (100 μM) also significantly reduced swim frequency (i: p < 0.001. n = 13) and burst durations (ii: p < 0.05. n = 5).

To test the extent to which endogenously released dopamine can inhibit swimming, the dopamine reuptake inhibitor bupropion (100 μM) was bath-applied (Fig. 3). The time to onset of bupropion effects were longer than those of dopamine or quinpirole (Fig. 3Aii), as might be expected, but within 20 to 30 minutes of application there was a clear reduction in spontaneous episode occurrence (Fig. 3Ai,Cii) and the range of inhibitory effects on swim parameters observed in the agonist experiments were replicated (Fig. 3B–D). The effects of bupropion were partially reversible upon return to control saline. These data strongly suggest that endogenously released dopamine, acting on D2-like receptors, can inhibit swimming in young *Xenopus* frog tadpoles.

### D4 receptor antagonism has excitatory effects on the swim network

**Figure 4:**
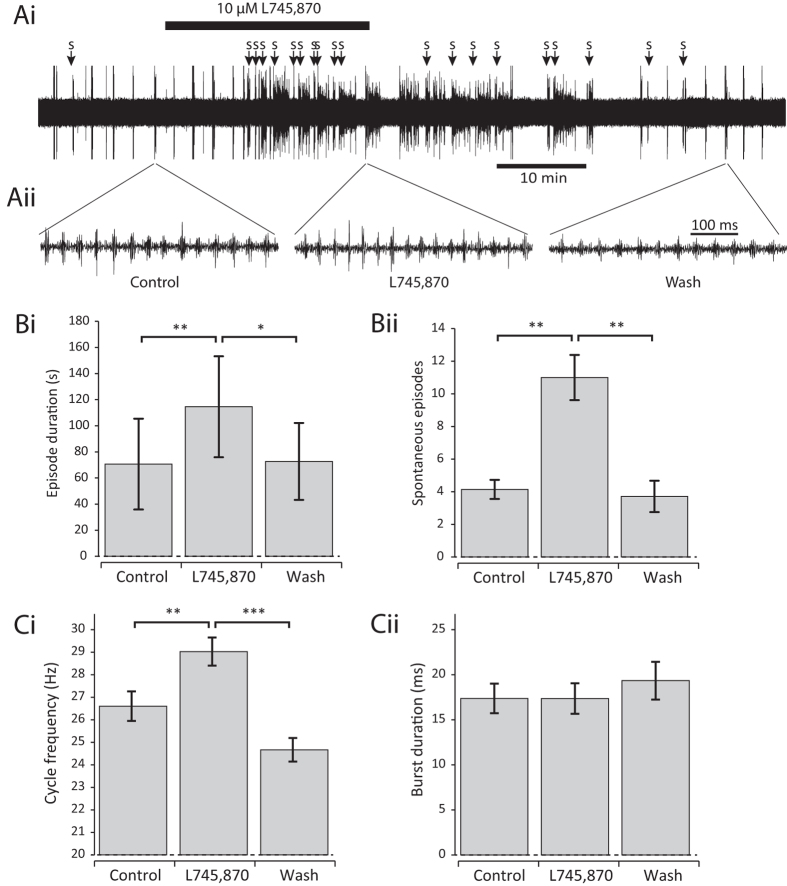
The D4 antagonist L745,870 has the opposite effects to dopamine and quinpirole. (**Ai**) Raw trace of fictive swimming showing an entire experiment. The application of 10 μM L745,870 has a clear excitatory effect on swimming. (**Aii**) Raw traces showing ~500 ms of activity at the start of an evoked episode in control, 10 μM L745,870, and following drug washout. B: L745,870 (10 μM) significantly increased episode duration (i: p < 0.01, n = 8) and the number of spontaneous episodes of swimming (ii: p < 0.01, n = 7). C. L745,870 (10 μM) also significantly reduced swim frequency (i: p < 0.01. n = 8) but had no significant effect on burst durations (ii: p > 0.05. n = 7).

Next we wanted to test whether dopamine inhibition is constitutively active. Amongst the inhibitory D2-like receptors (D2,D3,D4), the D4 receptor has been previously shown to play an especially important role in regulating spinal swim circuitry^10^. Therefore, we bath-applied the specific D4 receptor antagonist L745,870 (10 μM) and assessed its effects on fictive swimming. L745,870 caused a dramatic increase in the occurrence of spontaneous swim episodes (Fig. 4Ai,Bii). Moreover, swim episodes in the presence of L745,870 were both significantly longer in duration (Fig. 4Ai,Bi), and had a higher swim cycle frequency (Fig. 4Aii,Ci). We found no overall effect on burst duration (Fig. 4Cii). Overall, however, blockade of the D4 receptor results in the opposite effects to dopamine and quinpirole on most parameters of fictive swimming, suggesting that dopamine is constituitively released onto the *Xenopus* spinal cord where it acts, at least in part, via the activation of the D4 receptor.

### Cellular effects of D2-like receptor activation

**Figure 5:**
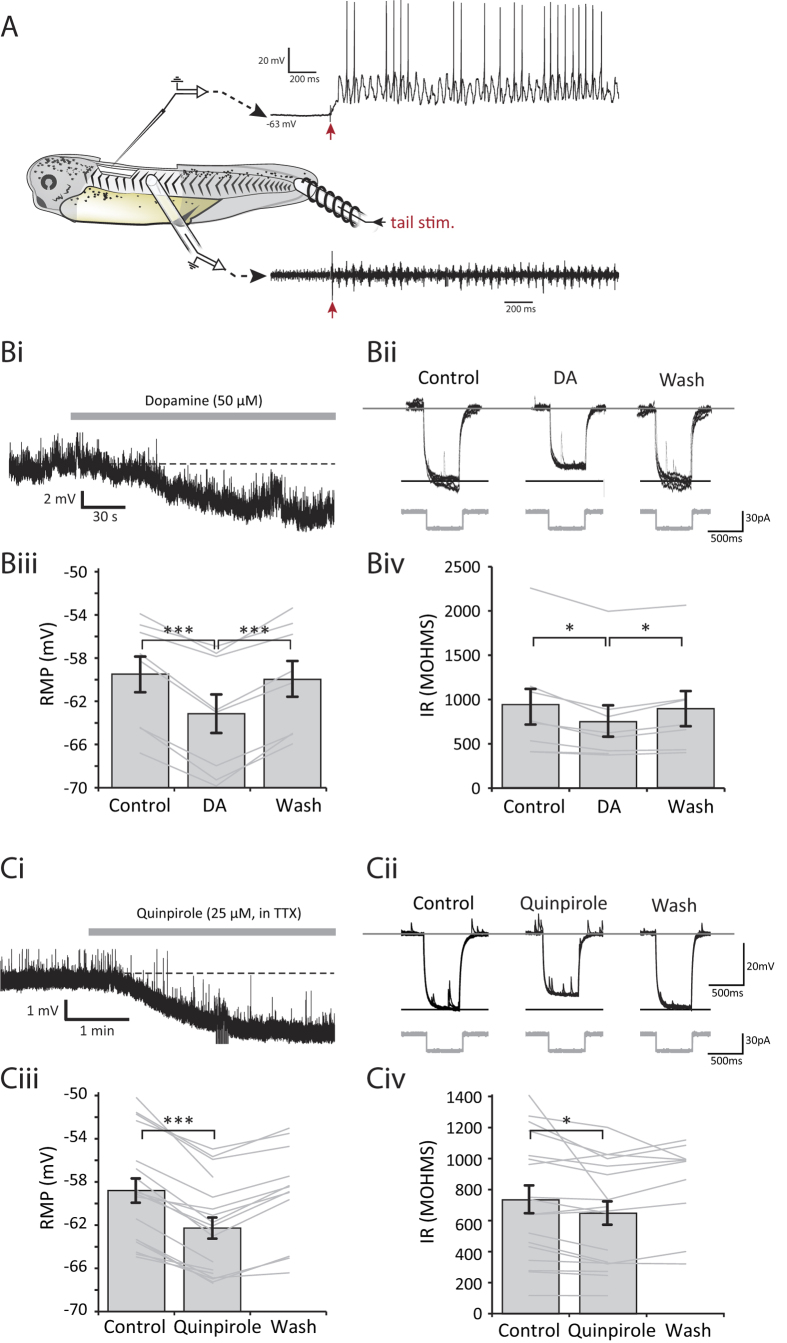
Activation of D2-like receptors hyperpolarises rhythmically active spinal neurons and decreases their input resistance. (**A**) The experimental preparation for making patch clamp recordings with simultaneous ventral root recordings. An intracellular recording of a rhythmically active spinal neuron is shown, with the ventral root trace shown below. The cell is the same as shown in (**Ci**). (**Bi**) Representative trace showing a hyperpolarisation caused by 50 μM dopamine in a rhythmically active spinal neuron. (**Bii**) Traces showing the membrane response to a 20 pA hyperpolarising current pulse under each condition. Six responses (sweeps) are overlaid for each condition. (**Biii**) Pooled data showing a significant hyperpolarisation of resting membrane potential in the presence of 50 μM dopamine (n = 8, p < 0.001) which significantly reversed upon washout (p < 0.001). (**Biv**) Pooled data showing a significant reduction in input resistance in the presence of dopamine (n = 8, p < 0.05) which significantly reversed upon washout (p < 0.05). (**Ci**) Representative trace showing a hyperpolarisation caused by 25 μM quinpirole in the presence of TTX. Note also the reduction in frequency of spontaneous synaptic potentials. (**Cii**) Traces showing membrane responses to a 20 pA hyperpolarising current pulse. Six responses (sweeps) are shown overlaid for each condition. (**Ciii**) Pooled data showing a significant hyperpolarisation in the presence of 25 μM quinpirole (n = 18, p < 0.001). (**Civ**) Pooled data showing a significant reduction in input resistance in the presence of quinpirole (n = 18, p < 0.05).

Having established that dopamine acts via an intrinsically active pathway on D2-like receptors in the spinal cord to profoundly inhibit fictive swimming, we next wanted to explore the cellular mechanisms responsible for this network inhibition. Therefore, patch clamp recordings were made from spinal neurons that are rhythmically active during fictive swimming (e.g., Fig. 5A). The most immediate effect of D2-like receptor activation was a rapid and reversible membrane potential hyperpolarization of spinal neurons. During the quiescent periods between swim episodes, dopamine (50 μM) reversibly hyperpolarized the membrane potential by up to 7 mV (Fig. 5Bi,Biii, mean change = 3.6 ± 0.4 mV; p < 0.001, n = 8). Furthermore, we found that this hyperpolarization was accompanied by an increase in membrane conductance, as evidenced by reduced voltage deflections in response to constant amplitude hyperpolarizing current pulses (Fig. 5Bii,Biv, p < 0.05, n = 8), which also significantly reversed upon washout. Quinpirole (25 μM) application similarly hyperpolarized the membrane potential (Fig. 5Ci,Ciii; mean change = 3.5 ± 0.3 mV; p < 0.001, n = 18), again accompanied by an increase in membrane conductance (Fig. 5Cii,Civ; p < 0.05, n = 18). The hyperpolarisation and decrease in input resistance induced by quinpirole persisted when applied in the presence of 1 μM TTX to block sodium spikes and therefore spike-mediated transmission (n = 5). This in turn suggests a direct postsynaptic effect of D2 receptor activation. Overall these data strongly suggest that the dopamine hyperpolarization of spinal neurons is mediated through the D2-like receptor-coupled opening of an ion channel, most likely a K^+^ channel.

### Evidence for the activation of a GIRK-like channel by dopamine

**Figure 6:**
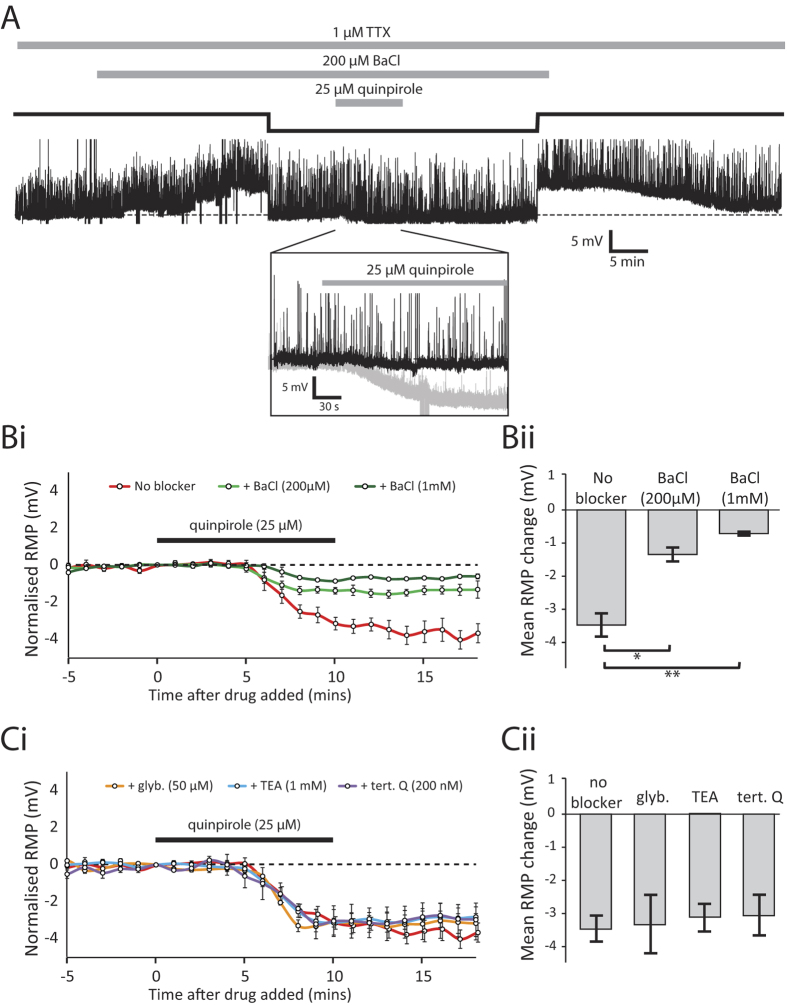
Barium chloride (BaCl), but not other potassium channel blockers, occludes the D2-like receptor mediated hyperpolarisation of spinal CPG neurons. (**A**) A representative intracellular recording showing the effects of 25 μM quinpirole in the presence of TTX and BaCl. Application of 200 μM BaCl depolarised the cell by approximately 4 mV before stabilising, at which point the membrane potential was corrected to control (dashed line) using DC current injection (solid black line). Quinpirole (25 μM) had only a small (<1 mV) hyperpolarising effect when subsequently applied in the presence of BaCl. The inset shows an expansion of the main trace. The effect of quinpirole alone is illustrated as a faded trace for comparison. (**Bi**) Timecourse showing the resting membrane potential, pooled and normalised into 1 minute bins, to illustrate the hyperpolarising effect of quinpirole (25 μM) under control conditions (red), in the presence of 200 μM BaCl (light green) and in the presence 1 mM BaCl (dark green). (**Bii**) Pooled data showing a significantly smaller hyperpolarisation in both 200 μM BaCl (p < 0.05, n = 4) and 1 mM BaCl (p < 0.01, n = 3) compared to control. (**Ci**) Timecourse plot showing the quinpirole hyperpolarisation in a range of other K^+^ channel blockers. (**Cii**) Various K^+^ channel blockers, including TEA, glybenclamide and tertiapin-Q showed no significant occlusion of the quinpirole effect.

To investigate the possibility that dopamine opens a K^+^ channel in spinal neurons, a range of pharmacological blockers of different K^+^ channel subtypes was applied in the presence of TTX to test which, if any, could occlude the subsequent quinpirole effect on membrane potential (Fig. 6). TEA had no effect on quinpirole’s ability to hyperpolarize spinal neurons (Fig. 6C, n = 3, p > 0.05), suggesting that voltage- and calcium-dependent K^+^ channels are unlikely to be involved. In contrast, barium chloride (BaCl), a broad spectrum blocker of inward-rectifying K^+^ channels, significantly occluded quinpirole’s effect on membrane potential in a dose-dependent manner (Fig. 6A,B). These data suggest that a member of the inward-rectifying family of K^+^ channels (K_ir_1-7) is likely to be responsible for the hyperpolarising effects of dopamine on membrane potential. Within this family of K^+^ channels, previous studies have shown that G protein-coupled, inward rectifying K^+^ channels (GIRKs) are a common target for D2-like receptors^16^. Therefore, we bath-applied tertiapin-Q, a highly selective blocker of GIRK1/4 heterodimer and ROMK1 (Kir_1.1_), but this failed to occlude the effects of quinpirole (Fig. 6C, n = 4, p > 0.05).

Because we were unable to directly antagonise the target channel we initially suspected to be involved in the quinpirole effects, we explored the possibility that another inward-rectifying channel may be involved. Alternative K_ir_ channels known to be targeted by dopamine are the ATP-sensitive K^+^ channels (K_ir_6) (e.g. refs 17 and 18). To rule these out as the target channel, we bath-applied glybenclamide (50 μM), a non-selective blocker of ATP-sensitive inward rectifier channels, but this had no effect on the quinpirole-induced hyperpolarization (Fig. 6C, p > 0.05, n = 3).

### Dopamine decreases the probability of neuron spiking during swimming

**Figure 7:**
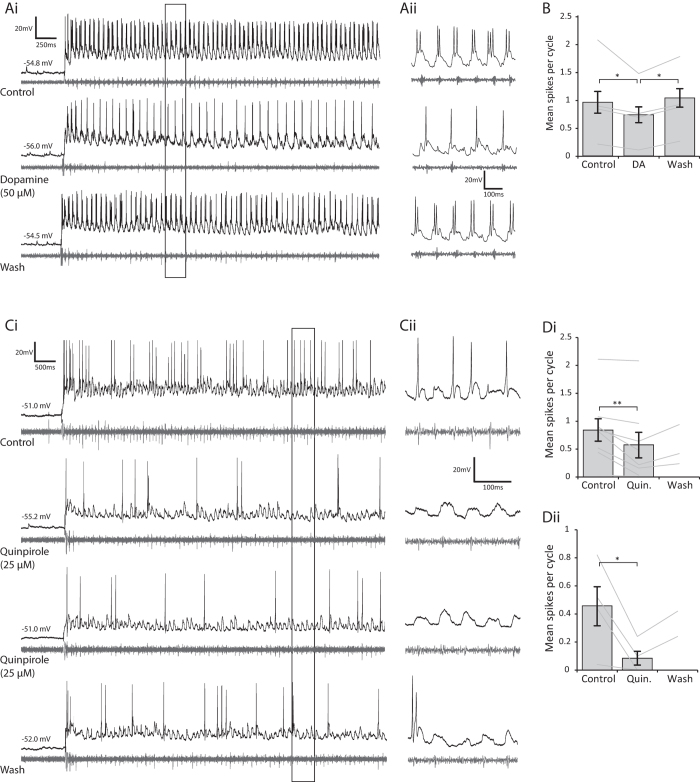
Activation of D2-like receptors leads to a reduction in spike reliability during swimming. (**Ai**) Raw traces showing an intracellular recording of a cIN (black) and below that the ventral root trace (grey) in control, in the presence of dopamine (50 μM) and following dopamine washout. (**Aii**) The inset shows an expansion of the trace in Ai as indicated by the black box. (**B**) Pooled data shows that 50 μM dopamine significantly reduced the reliability of spiking during swimming (p < 0.05, n = 7), which reverses significantly upon washout of dopamine. (**Ci**) Raw trace of a recording from an aIN in control, in the presence of quinpirole (10 μM) both without and with corrected RMP, and following quinpirole washout. (**Cii**) The inset shows 250 ms of activity from the black box in Ci. Pooled data shows that quinpirole significantly reduced the reliability of spiking during swimming both without (**Di**, p < 0.01, n = 8) and with corrected RMP (**Dii**, p < 0.05, n = 4).

Next we wanted to explore the impact of D2 receptor-mediated changes in membrane potential and conductance upon neuronal firing properties. During bouts of swimming, many rhythmically active spinal neurons, with the exception of the rhythm-generating dINs, can vary in the number of spikes they generate in each swim cycle, with important consequences for the temporal dynamics of overall network output^19^. Thus, we first checked whether neuron spike probability was affected when swimming was evoked following D2-like receptor activation. Dopamine (50 μM) caused a significant reduction in the firing probability of rhythmically active spinal neurons during swimming (Fig. 7A,B, n = 7, p < 0.05), which reversed upon washout of the drug. The same effect was found for quinpirole (10–25 μM), which also caused a clear and significant decrease in spike reliability during swimming (Fig. 7C,D n = 8, p < 0.01). This effect was observed across a range of rhythmically active cell types including motorneurons (mns: n = 3 confirmed), commissural interneurons (cINs: n = 6 confirmed, e.g. Fig. 7A) and ascending interneurons (aINs: n = 3 confirmed). The firing probability remained lower during swimming even when the resting membrane potential of the neuron was artificially brought back to the pre-quinpirole value using tonic DC current injection (Fig. 7C, n = 4, p < 0.05), suggesting that D2-like receptor activation affects presynaptic inputs to the recorded neurons and/or that other properties of the neuron have been affected (such as rheobase). The effects of quinpirole were significantly harder to reverse than dopamine and did not always wash within the timescale of our experiments; however, on occasions when recordings were maintained for longer periods (>1 hour) the effects eventually washed off.

### Dopamine affects the intrinsic properties of spinal neurons

**Figure 8:**
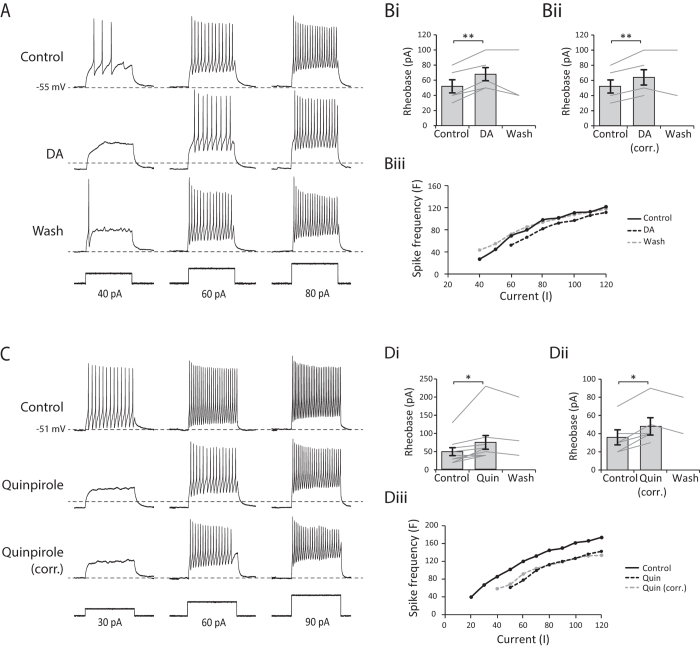
The activation of D2 receptors modifies the integrative electrical properties of spinal CPG neurons. (**A**) An example of the cellular response to depolarising current pulses in control, in 50 μM DA and following washout of dopamine. (**Bi**) Pooled data showing that dopamine significantly increases rheobase (p < 0.01, n = 5). (**Bii**) The effect persists when the RMP of the cell is corrected to control value using DC current injection (p < 0.01, n = 5). (**Biii**) F-I plot for the cell shown in A. Dopamine shifted the FI curve to the right, demonstrating reduced firing frequency in response to the same current input. (**C**) Responses to depolarising current pulses in control, in 25 μM quinpirole and following washout of quinpirole. (**Di**) Pooled data showing that quinpirole also significantly increases rheobase (p < 0.05, n = 9), which persisted when the RMP was corrected (**Dii**, p < 0.05, n = 5). (**Diii**) F-I plot for the cell shown in (**C**). Quinpirole shifted the FI curve to the right, demonstrating reduced firing frequency in response to the same current input.

A reduction in firing reliability during swimming can be explained either by changes in the integrative properties of the recorded neurons, or in the efficacy of synaptic inputs from neurons elsewhere in the network, or both. To test between these hypotheses we used a protocol in which a series of depolarising current pulses (200 ms duration) with increasing amplitude (steps of 10 pA) was injected into neurons to assess any dopaminergic effects on their intrinsic excitability. In dopamine (50 μM), a larger amplitude current pulse was required to evoke an action potential (Fig. 8A,Bi, n = 5, p < 0.01). Moreover, this was not simply due to the RMP being further away from spike threshold because we found the same effect when the RMP was corrected back to the control level using DC current injection (Fig. 8Bii, n = 5, p < 0.01). In a number of neurons, we also observed an effect on the firing frequency in response to current injection. In 3 of 5 cells there was a clear rightward shift in the F-I relationship, indicating a decrease in neuronal excitability characterised by a reduction in spike frequency in response to the same current input (e.g. Fig. 8A,Biii). As in previous experiments, we found the same effects for quinpirole (10–25 μM), with a larger amplitude current pulse needed to induce an action potential in the presence of the drug (Fig. 8C,Di, n = 9, p < 0.05), which persisted when the RMP was corrected back to control (Fig. 8Dii, n = 5, p < 0.05). In 6 of 9 cells we found a rightward shift in the F-I relationship (e.g. Fig. 8C,Diii). This general reduction in firing probability persisted when the RMP was corrected back to the control level. Consistent with our extracellular data, these results suggest that the activation of D2-like receptors leads to a reduction in the excitability of rhythmically active spinal neurons.

## Discussion

Dopamine is a widely distributed and phylogenetically conserved modulator of central neural circuits. In vertebrates, the dopamine system has long been associated with motor control, largely due to the Parkinsonian symptoms that accompany the loss of dopaminergic neurons in the substantia nigra^20^. However, there is also growing evidence that descending pathways to the spinal cord release dopamine to modify ongoing locomotor output produced by spinal CPGs (see ref. 21 for a recent review). In the spinal cord, dopamine acts on receptors belonging to two broad classes: D1-like receptors, which are typically excitatory; and D2-like receptors, which are typically inhibitory. In free swimming larval stages of *Xenopus laevis,* dopamine has opposing actions depending upon which of these receptor classes is activated: low dose dopamine selectively activates higher affinity D2-like receptors to reduce spontaneous swim bout occurrence, while higher doses activate lower affinity D1 receptors to increase swimming^6^. The present study sought to address two related and unanswered questions regarding dopamine modulation: firstly, what are the effects of dopamine on swimming at earlier stages of *Xenopus* development when much more is known of the CPG circuit and when the tadpole is normally sessile until stimulated to swim; and secondly, what are the cellular consequences of dopamine receptor activation in this model system? Our extracellular ventral root recordings show that dopamine, acting solely via D2-like receptors at all concentrations tested, exerts strong inhibitory effects on the full range of locomotor parameters including the occurrence of spontaneous swimming, episode duration as well as the frequency, duration and amplitude of individual locomotor bursts (Figs 1 and 2). Moreover, consistent with studies in zebrafish^5^, and lamprey^22^,^23^, the application of bupropion to block the reuptake of dopamine potently mimicked these inhibitory effects on the swim network (Fig. 3), suggesting that the effects of dopamine constitute a behaviourally relevant and functioning modulatory system. In support of this hypothesis, we found that antagonising the D4 receptor led to a block of inhibition, leading to excitatory effects on the swim network (Fig. 4). Interestingly, a low concentration of dopamine had no effect on burst durations, whereas a high concentration of dopamine significantly reduced burst durations. All other parameters of swimming were inhibited at both concentrations, and all parameters were inhibited by the D2-like agonist quinpirole. One possible explanation for the lack of effect of low dopamine on burst durations is that different subtypes of the D2-like receptor are expressed in motoneurons (to affect burst durations) and interneurons (to affect rhythm frequency). For instance, dopamine binds with a higher affinity to the D4 (and D3) receptor compared to the D2 receptor^24^. This raises the possibility that the low concentration of dopamine may act upon D4 receptors in interneurons, but not D2 receptors in motoneurons, which are only activated by the higher dopamine concentration. In support of this explanation, the highly specific D4 receptor antagonist L745,870 had excitatory effects on all the swim parameters that were affected by dopamine *except* for burst durations.

The effects of dopamine on a rhythmic network vary according to the species^2^; the developmental stage^5^; the drug concentration^6^; and even the cell type being targeted^25^. This heterogeneity of action has often made the precise role of dopamine in rhythmic networks difficult to define. In zebrafish, dopaminergic input to the spinal cord is known to derive exclusively from the descending dopaminergic diencephalic tract (DDT)^26^, which plays diverse roles in establishing the complement of spinal CPG cell types, in modulating ongoing swimming, and controlling developmental changes in swim pattern^5^,^9^,^10^,^11^. Dopamine modulation of mammalian locomotor networks is also, unsurprisingly, complex, in part due to the heterogenous patchwork of receptor subtypes (including D1-D5) expressed in the ventral horn of the spinal cord^2^,^21^, but also due to differences in the effects of dopamine in different preparations^27^. For drug-induced locomotor activity in rodents, dopamine slows the bursting rhythm through actions on D2-like receptors while stabilising the rhythm through D1-like receptors^28^,^29^. The mechanisms are not fully understood but in mice, at least, dopamine increases the excitability of motorneurons via a D1 receptor mediated depolarisation and a decrease in at least two potassium conductances: I_A_ and SK_CA_^7^.

Various lines of evidence from our intracellular recordings suggest that in *Xenopus* tadpoles, dopamine acts primarily through the D2-like receptor coupled opening of a K^+^ channel expressed heterogeneously on various spinal neuron types (Figs 5 and 6). We consistently found a TTX-resistant membrane potential hyperpolarisation with relatively fast onset that was associated with a decrease in input resistance, consistent with the opening of either a chloride, or more likely, a potassium channel. We ruled out most voltage-dependent K^+^ channels, another common target for dopamine, as TEA was unable to reduce the hyperpolarisation. Barium chloride, on the other hand, significantly occluded the hyperpolarisation in a dose-dependent manner, strongly suggesting an inward-rectifying K^+^ channel. However, two blockers of specific inward-rectifier K^+^ channels, tertiapin-Q (GIRK channel blocker), and glybenclamide (ATP-dependent K^+^ channel blocker) were unable to occlude the quinpirole hyperpolarisation. Therefore, with the current data we are unable to determine the exact K^+^ channel subtype involved. Although TEA had no significant effect on the hyperpolarisation of spinal neurons, it is possible that other BaCl-sensitive, TEA-insensitive potassium channels, such as flickering potassium channels^30^ or Na^+^ -dependent potassium channels^31^, may be the target. It would be interesting in future studies to explore whether the mechanism here involves the canonical AC/cAMP pathway, or whether a membrane-delimited coupling to GIRK channels is involved. Overall, we favour the latter hypothesis, as GIRK channels are a highly common target for D2-like receptors across a range of tissue types^32^; the antagonist L745,870 used in this study is known to block constitutively active GIRK currents^33^; and finally, the onset of effects are an order of magnitude faster than modulators in this system that involve second messenger cascades (serotonin and noradrenaline), suggesting a direct coupling between D2 and the target channel^14^. On the other hand, the GIRK blocker tertiapin-Q failed to occlude the hyperpolarisation. However, tertiapin-Q is a highly selective blocker of the GIRK1/4 heterodimer^34^, whereas neuronal GIRK channels are comprised of homo- or hetero-tetramers containing GIRK1–GIRK3 subunits^35^. It is possible that a dimer involving GIRK 2 or 3, or indeed a homologous amphibian GIRK channel that is insensitive to tertiapin-Q, may be involved in dopamine modulation of *Xenopus* swimming.

A hyperpolarisation caused by the opening of a K^+^ channel shifts neurons away from firing threshold, and hence they showed reduced spiking during swimming and in response to current injection (Figs 7 and 8). These effects persisted even when the membrane potential was corrected for by tonic DC current injection, thus the accompanying conductance increase is also likely to shunt the membrane and reduce the responsiveness of neurons to excitatory inputs. Indeed, a decrease in input resistance is known to shift F-I curves to the right^36^. It is possible that these effects on firing could be due to an indirect effect mediated by the activation of D2-like receptors located elsewhere in the network. However, we think this is unlikely as the hyperpolarisation and decrease in input resistance persisted in TTX, suggesting a direct effect on CPG neurons. Moreover, we know that D2-like receptors are located in the spinal cord as the effects of dopamine persist even when the brain is removed. It is possible, even in TTX, that the effects on recorded cells could be propagated via gap junction coupling^37^. Again, however, we think this is unlikely, as electrical coupling is thought to be restricted to the dIN^38^ and motoneuron^39^ populations, whereas we observed consistent effects in a range of CPG neuron types.

Can the effects of dopamine on overall network output be explained by the effects on the spiking properties of specific spinal neuron subtypes? In the lamprey, dopamine (10–100 μM) decreases the frequency of glutamate-induced fictive swimming through the D2 receptor-mediated depression of low-voltage activated (LVA) calcium channels on spinal commissural interneurons (cINs)^4^,^22^,^23^,^40^. These LVA channels contribute to the post-inhibitory rebound (PIR) property of cINs which normally hastens the onset of swim cycles, so their inhibition by dopamine slows swimming. Here we also observed that dopamine had a strong inhibitory effect on both swim frequency and the cINs (e.g. cell in Fig. 7Ai). A reduction in cIN firing in *Xenopus* tadpoles is known to reduce PIR in the descending interneuron (dIN) population, also slowing the onset of the next bout of excitatory drive, slowing swim frequency^41^. For the motorneuron population, the reduction in firing we observed during swimming leads to a reduction in the duration of locomotor bursts and in turn reduced excitatory output to axial swim muscles^19^.

In contrast to older free-swimming *Xenopus* tadpoles^6^, we found no evidence for an excitatory, D1-mediated effect of high dopamine concentration, suggesting that developmental changes in *Xenopus* motor behaviour may be mediated by an increase in D1 receptor expression. Although a systematic, developmental analysis of dopamine receptor expression is yet to be performed in *Xenopus* tadpoles, findings from other model systems support this overall developmental pattern of receptor expression. For example, a recent study in mice showed that high levels of inhibitory D2-like receptors are found in the spinal cord throughout development, whilst excitatory D1 receptor expression is low at early stages but increases with development, leading to an age-dependent increase in excitatory drive in the spinal cord^42^. Similarly, there is abundant expression of inhibitory D2 receptors in the motoneurons and interneurons of young larval lamprey^43^, which remains high in the adult lamprey^44^, although less is known about spinal D1 receptor expression in this system. In addition to the developmental onset of this D1 excitation in *Xenopus* tadpoles, the mechanism of excitation by D1 activation is also an avenue for future research. Interestingly, barium chloride depolarised spinal neurons by ~4 mV (Fig. 8A), suggesting that the suspected target K^+^ channels are likely to be open at rest. This is supported by the excitatory effects of the D4 receptor antagonist on the swim network. Thus, one possibility is that these K^+^ channels become a target for the later developing spinal D1 receptors, which may close, rather than open them.

In summary, we show that dopamine acts at these early stages of development in *Xenopus* tadpoles as a potent inhibitory modulator of the locomotor network controlling swimming, and acts through the D2-like receptor coupled hyperpolarisation of CPG neurons in the spinal cord. During development, this inhibitory D2-like pathway persists^6^,^45^, but at some developmental stage, likely around the onset of free-feeding and free-swimming^46^, there is a switch to excitation by high dopamine concentrations, presumably through the introduction of excitatory D1-like receptors to the swim network.

## Methods

### Experimental animals

All experiments conformed to UK Home Office regulations and were approved by the Animal Welfare Ethics Committee (AWEC) of the University of St Andrews. All experiments were performed on newly hatched pre-feeding *Xenopus laevis* tadpoles at developmental stage 37/38 or 42^47^. Tadpoles were reared from fertilized ova obtained following breeding of adults selected from an in-house colony. Mating was induced by injections of human chorionic gonadotropin (HCG, 1000 U/ml, Sigma, UK) into the dorsal lymph sac of breeding pairs of adult frogs.

### Electrophysiology

*Xenopus* tadpoles were immobilized by gashing the skin before placing in 12.5 μM α-bungarotoxin saline for approximately 30 minutes, and then mounted on a rotatable Sylgard platform in a bath of saline (in mM: 115 NaCl, 2.5 KCl, 2 CaCl_2_, 2.4 NaHCO_3_, 1 MgCl_2_, 10 HEPES, adjusted with 4 M NaOH to pH 7.4). Both sides of the trunk skin overlying the myotomal muscles were removed using a finely etched needle and forceps. The dorsal parts of approximately 7 rostral myotomes were freed from the spinal cord and the roof of the hindbrain and spinal cord was opened to the neurocoel to improve drug access and provide access for patch clamp electrodes. For whole-cell current clamp recordings, exposed neuronal somata were patch clamped using borosilicate glass pipettes (Harvard Apparatus Ltd) pulled on a Sutter P97 pipette puller. Patch pipettes were filled with 0.1% neurobiotin (Vector lab) in the intracellular solution (in mM: 100 K-gluconate, 2 MgCl_2_, 10 EGTA, 10 HEPES, 3 Na_2_ATP, 0.5 NaGTP adjusted to pH 7.3 with KOH) and had resistances of ~10 MΩ. Changes in membrane potential were recorded in current clamp mode using an Axoclamp 2B amplifier. Simultaneous extracellular recordings of fictive swimming were made with suction electrodes from ventral roots at intermyotomal clefts, and signals were amplified using differential AC amplifiers (A-M Systems Model 1700). Simultaneous intracellular and extracellular signals were digitized using a CED Power 1401, and displayed and stored on a PC computer using Spike 2 software. Fictive swimming was initiated by stimulating through a glass suction electrode placed on the tail skin, which delivered a 1 ms current pulse via a DS2A isolated stimulator (Digitimer).

### Pharmacological agents

All drugs were obtained from Sigma-Aldrich or Tocris Bioscience and bath-applied to the preparation. Dopamine hydrochloride (Sigma) was always made up in distilled H_2_0 prior to the experiment (<1 hour) and stored on ice in the dark to minimise oxidation. All other drugs were made up to stock concentrations and frozen in aliquots, defrosted before an experiment, and diluted to final concentrations. Quinpirole hydrochloride (Tocris) and Bupoprion hydrochloride (Tocris) were both dissolved in distilled H_2_0. L745,870 trichydrochloride (Tocris) was dissolved in DMSO.

### Data analysis

Electrophysiological data were first analysed using dataview software (v 10.3.0, courtesy of Dr. W. J. Heitler) and all raw data were imported into Excel spreadsheets and analysed. Statistical analyses were conducted using PASW statistics 21. A rest time of 2 minutes was given between evoked episodes of swimming to ensure each episode was not influenced by preceding activity^48^,^49^. For swim episode duration analysis we calculated a mean of 3 consecutive evoked episodes in each condition. For intra-episode swim parameters (cycle frequency, burst duration, burst amplitude) a mean of the first 20 cycles of swimming across 3 episodes was calculated for each condition. For analysis of spontaneous swimming we calculated the number of spontaneously occuring swim episodes in a 20 min period in each condition. To calculate spike probability, we measured the number of action potentials in the first 50 cycles of swimming in an episode and divided this value by the number of cycles. For all experiments, values are displayed as mean ± SEM and unless otherwise stated conditions were compared using either paired t-tests or repeated measures ANOVAs followed by Bonferonni-corrected post-hoc comparison. For patch clamp data where we obtained a wash value in only a subset of experiments, only the individual wash values are displayed but not the mean value.

### Neuron identification

Following patch-clamp recordings, animals were fixed in 2% glutaraldehyde in 0.1 M phosphate buffer, pH 7.2, overnight in a refrigerator (∼4 °C). Animals were first rinsed with 0.1 M PBS (120 mM NaCl in 0.1 M phosphate buffer, pH 7.2), and washed in two changes of 1% Triton X-100 in PBS for 15 min with agitation. Next, animals were incubated in a 1:300 dilution of extravidin peroxidase conjugate in PBS containing 0.5% Triton X-100 for 2–3 hours with agitation, and washed again in at least four changes of PBS. Animals were then immersed in 0.08% diaminobenzidine in 0.1 M PBS (DAB solution) for 5 min, moved to a DAB solution with 0.075% hydrogen peroxide for 1–2 min, and then washed in running tap water. Finally, animals were dehydrated in 100% alcohol, cleared in methyl benzoate and xylene, and mounted whole between two coverslips using Depex. Neuronal cell bodies and axon processes were observed under a x40 objective to identify CPG neuron types. All reagents were obtained from Sigma or Tocris Bioscience.

## Additional Information

**How to cite this article**: Picton, L. D. and Sillar, K. T. Mechanisms underlying the endogenous dopaminergic inhibition of spinal locomotor circuit function in *Xenopus* tadpoles. *Sci. Rep.* **6**, 35749; doi: 10.1038/srep35749 (2016).
